# Corticosteroids as adjunctive therapy in patients with acute/subacute paracoccidioidomycosis presenting a severe paradoxical inflammatory reaction: Two case reports and literature review

**DOI:** 10.1016/j.bjid.2025.104608

**Published:** 2026-01-13

**Authors:** Pedro Stringelli-Brandão, Marília Prior Fuga, Márcio Ketner Sguassábia, Ivonete Helena Rocha, Mario León Silva-Vergara

**Affiliations:** Universidade Federal do Triângulo Mineiro, Internal Medicine Department, Infectious Diseases Unit, Uberaba, MG, Brazil

**Keywords:** Acute/subacute PCM, Corticosteroids, Paradoxical inflammatory reaction, IRIS

## Abstract

Paradoxical inflammatory reactions, similar to Immune Reconstitution Inflammatory Syndrome (IRIS) in HIV, have been occasionally reported in leprosy, tuberculosis, certain immunosuppressive conditions, and Paracoccidioidomycosis (PCM), among others. This report presents the clinical data and outcomes of two PCM cases in which the patients developed a severe inflammatory reaction following antifungal therapy, with subsequent improvement after adjunctive corticosteroid use. Both patients were adults with acute/subacute PCM who experienced clinical worsening after starting liposomal amphotericin B, with fever, anasarca, jaundice, and exacerbation of pre-existing symptoms. After excluding other infections, intravenous hydrocortisone was administered, resulting in rapid improvement. Corticosteroids were tapered after two to three weeks, and both patients continued outpatient follow-up while receiving itraconazole. Few similar PCM cases have been described in the literature, and they reported comparable outcomes. Its exact mechanism remains unclear, but may be immune-mediated. Reporting additional cases is essential to better establish the true incidence of this reaction and to strengthen the evidence supporting the benefit of corticosteroids as an adjunctive therapy.

## Introduction

Paracoccidioidomycosis (PCM) is a neglected fungal disease endemic in Latin America in which ten million people can be infected by five of the thermodimorphic fungi species belonging to the genus *Paracoccidioides*. The infection occurs through the inhalation of fungal propagules present in the environment and most individuals remain asymptomatic throughout their lives. Its real occurrence is unknown and has been estimated in 1‒4 cases per 100.000 inhabitants^1^, ^2^, ^3^

Most cases of PCM occur in male adults who live or engage in activities in rural areas of endemic regions. This disease presents a broad spectrum of clinical and immunological features, and two clinical forms are well recognized. The acute/subacute form, which predominantly affects children, adolescents, and young adults, represents 20 %–25 % of PCM cases. It occurs a few weeks to months after infection and involves organs of the mononuclear phagocyte system, as well as the skin and bones. In contrast, the chronic form affects adult males who typically present with pulmonary and mucosal lesions, although other organs may also be involved^4^^,^^5^

Patients with the acute/subacute form of PCM exhibit specific cell-mediated immunosuppression induced by fungal antigens. In this clinical form, involvement of the mononuclear phagocyte system explains the severity and rapid progression observed in most cases^5^ The use of corticosteroids in patients with severe PCM has raised concern over the years, especially when an intense inflammatory reaction triggered both by the fungus and by the specific therapy occurs, which can lead to multiple organ dysfunction and tissue damage. They have been prescribed for short periods in PCM patients presenting with ocular, cerebral, and laryngeal involvement, chylous ascites, and fungal septic shock, among other manifestations, with reported clinical improvement^6^, ^7^, ^8^

This paradoxical inflammatory reaction following antifungal therapy has been described in a few cases of the acute/subacute or disseminated clinical forms of PCM, in which patients received corticosteroids as an adjunctive measure and experienced remarkable clinical benefit^6^^,^^9^^,^^10^ This reaction is similar to IRIS observed in HIV-infected patients with advanced immunodeficiency several weeks or months after starting Antiretroviral Therapy (ART). It has also been described in non-HIV patients with lepromatous leprosy, in those with pulmonary or extrapulmonary tuberculosis following specific therapy, and in transplant recipients infected with *Cryptococcus neoformans* after withdrawal of immunosuppression^11^, ^12^, ^13^, ^14^

Most of these patients improved after a course of corticosteroids as an adjunctive measure and continued the specific therapy. Currently, the mechanisms involved in this reaction are still unclear, and according to several authors, they may be immune-mediated when massive antigen release occurs after bacterial, viral, or fungal death. Partial immune restoration following the suspension or introduction of specific therapies could also trigger this paradoxical inflammatory reaction^5^^,^^15^^,^^16^ The aim of this report is to present clinical data and outcomes of two acute/subacute PCM cases that developed similar severe paradoxical inflammatory reactions following antifungal therapy and who improved with corticosteroids as an adjunctive measure.

A signed consent form was obtained from both patients.

## Cases presentation

### Patient 1

A 54-year-old Brazilian female housewife was admitted to the teaching hospital in late March 2024 with a four-month history of daily fever, generalized lymphadenomegaly, asthenia, hyporexia, abdominal distension with persistent diffuse abdominal pain, early satiety, nausea, vomiting, and a 16 kg weight loss. She was prediabetic, a heavy smoker, and denied a family history of cancer.

Earlier, she had been evaluated by the surgical team and, on clinical examination, presented with fever, cutaneous-mucosal pallor, and retroauricular, cervical, axillary, supraclavicular, and inguinal lymph nodes of varying sizes, with elastic consistency and inflammatory signs. In addition, the abdominal examination showed marked hepatosplenomegaly and non-painful epigastric and mesogastric masses.

She underwent an upper endoscopy, which showed pangastritis and duodenal ulcers. The abdominal CT revealed hepatosplenomegaly and para-aortic, perigastric, and mesenteric coalescent lymph nodes, whereas the chest CT demonstrated mediastinal and bilateral hilar lymphadenomegaly. Based on these findings, a suspicion of lymphoma was raised, and a CT-guided needle biopsy was performed. The histopathological examination of the retroperitoneal lymph node fragment revealed a granulomatous reaction with birefringent yeasts showing multiple budding, compatible with *Paracoccidioides* spp. Serological tests for hepatitis A, B, and C, as well as for Human Immunodeficiency Virus (HIV), were negative. Additional laboratory tests performed at admission are presented in Table 1.

**Table 1:** Laboratorial evaluation at admission.

	Patient 1	Patient 2
**Hemoglobin (g/dL)**	8.2	9.2
**White Blood Cells**	10.590	12.500
**Eosinophils (%)**	14.4	3.6
**Platelets**	513.000	330.000
**Albumin (g/dL)**	2.5	3.3
**Ferritin (ng/mL)**	658.16	3.543,33
**CRP (mg/L)**	13.611	41.473
**Alkaline Phosphatase (UI/L)**	1.107,7	298.2
**GGT (UI/L)**	1.366,8	204.8
**AST (UI/L)**	238.7	31.8
**ALT (UI/L)**	90.2	36.3

GGT, Gamma-Glutamyl Transferase; AST, Aspartate Aminotransferase; ALT, Alanine Aminotransferase; CRP, C-Reactive Protein.

She was then admitted to the Infectious Diseases Unit (IDU) in April 2024 with a diagnosis of the acute/subacute clinical form of PCM, and a daily dose of liposomal amphotericin B at 5 mg/kg was initiated. Despite 11-days of antifungal therapy, her clinical condition worsened. She developed severe anasarca, her abdominal circumference increased from 90 cm to 105 cm, and jaundice, vomiting, diarrhea, and dyspeptic symptoms either appeared or were exacerbated. At this point, parenteral nutrition was required for one week. After other infections were ruled out, intravenous hydrocortisone at 300 mg/day was prescribed on day 13, resulting in progressive clinical improvement. Two weeks later, the corticosteroid was tapered. Antifungal therapy was maintained.

Because she showed clinical and laboratory improvement, she was discharged after forty days for outpatient follow-up on itraconazole 200 mg twice daily. During the first months, there was complete regression of the lymphadenopathy, although moderate ascites and abdominal discomfort persisted, which led to her readmission five months later. A diagnostic/therapeutic paracentesis was performed, and chylous peritoneal fluid was identified. After the procedure, there was significant improvement in her abdominal complaints, with no need for further interventions. She is currently asymptomatic while on itraconazole therapy.

### Patient 2

A 28-year-old Brazilian male security guard was admitted to the teaching hospital with a history of fever, abdominal pain, nausea, vomiting ‒ especially after meals and drinks ‒ and an unintentional weight loss of 30 kg over two months. He had previously been admitted to another hospital with the same symptoms, and acute pancreatitis was diagnosed. He remained hospitalized for eight days and, after clinical improvement, was discharged. Due to the recurrence of symptoms, he was referred to the teaching hospital for further clinical evaluation.

At admission, he was first evaluated by the surgical team due to the previous diagnosis of acute pancreatitis. Physical examination revealed an obese patient (weight of 105 kg) with mucocutaneous pallor, moderate jaundice, diffuse abdominal pain, hepatosplenomegaly, and enlarged, painful lymph nodes with firm consistency located in the retroauricular, suboccipital, and axillary regions. No signs of peritonitis or pancreatitis were observed.

The abdominal CT showed coalescent retroperitoneal and mesenteric lymph nodes, hepatosplenomegaly, and mild ascites. Based on these findings, a suspicion of lymphoma arose. However, the histopathological examination of a cervical lymph node fragment revealed a chronic granulomatous reaction with birefringent yeasts exhibiting multiple budding, suggestive of *Paracoccidioides* spp. Serological tests for HIV and hepatitis A, B, and C were negative. Other laboratory results are presented in Table 1.

Liposomal amphotericin B at a daily dose of 5 mg/kg was then initiated, and five days later, he presented clinical worsening, with anasarca, severe jaundice, prostration, deterioration of his general condition, and a rapid decrease in hemoglobin levels, despite no evidence of bleeding. As other infections were ruled out, hydrocortisone 300 mg/day was prescribed. The patient showed significant improvement in symptoms, and corticosteroids were tapered three weeks later. Antifungal therapy was maintained. He remained hospitalized for an additional three weeks and was then discharged for outpatient follow-up. He is currently asymptomatic while on itraconazole 200 mg twice daily.

## Discussion

After more than one century since its first description in Brazil, PCM remains a neglected disease. Its prevalence, as well as its social and medical costs ‒ including hospitalization, prolonged antifungal therapy, pulmonary and abdominal fibrosis and related complications, aesthetic scarring of mucosae, school and work absenteeism, among others ‒ remain completely unknown. Moreover, most patients living in endemic areas must overcome economic and social barriers to access medical care where they can be properly and promptly diagnosed and treated^1^^,^^3^^,^^17^

Among the two classical clinical forms of PCM, the acute/subacute form accounts for 20 %–25 % of cases and evolves rapidly toward systemic involvement, presenting polymorphic and severe clinical manifestations with poor outcomes when early diagnosis and specific therapy are delayed. In contrast, the chronic form affects adult males during their productive years, causing pulmonary and mucosal lesions and may eventually become disseminated and severe. Notably, PCM represents the eighth leading cause of mortality due to chronic infectious diseases in Brazil^4^^,^^17^, ^18^, ^19^

This report presents clinical and outcome data of two adult patients with the severe acute/subacute clinical form of PCM. Due to the rapid deterioration of their clinical and laboratory parameters, and the development of a paradoxical inflammatory reaction after several days of antifungal therapy, corticosteroids were introduced as adjunctive treatment. Both patients showed remarkable clinical improvement, and after a few weeks, corticosteroids were discontinued without recurrence of symptoms. Notably, both patients lived in Uberaba, Minas Gerais, where autochthonous cases of the acute/subacute form have been diagnosed in individuals without a history of rural exposure, despite the presence of animal husbandry areas, woodlands, and farms around the city that create a rural-like environment conducive to infection.

The first patient developed chylous ascites during follow-up, which improved after a diagnostic/therapeutic paracentesis. This is a rare complication of PCM resulting from an inflammatory process with lymph node fibrosis, which may lead to lymphatic obstruction and abdominal and/or pleural leakage of lymph. In another similar case previously reported, corticosteroids were used without benefit^7^

The antigen-specific immunosuppression observed in PCM patients is reversed during antifungal therapy and may be related to the paradoxical inflammatory reaction, which has been rarely reported^6^^,^^9^^,^^10^ The seven similar cases previously described, together with the two cases presented herein, are summarized in Table 2. Most patients exhibited the severe acute/subacute clinical form of PCM, were male, received corticosteroids for a median duration of 41.1 days, and demonstrated clear clinical benefit from this intervention. As suggested by others, its use must be limited to a short period of time, including the time required for tapering the drug^6^

**Table 2:** Update of the reported cases of paracoccidioidomycosis presenting an inflammatory paradoxical reaction who received corticosteroids as adjunctive therapy.

References	Number of Patients	Age (years)	Gender	Symptoms duration (months)	Clinical Form	Anatomical Site Affected	Corticotherapy	Time (days)	Clinical Decision	Outcome
Gryschek et al., 2010	2	14	M	NA	Acute/Subacute	Lymph nodes	Prednisone	112	Paradoxical inflammatory reaction	Improvement
		12	M	NA	Acute/Subacute	Lymph nodes	Prednisone	63	Paradoxical inflammatory reaction	Improvement
		61	M	6	Chronic	Brain	Dexamethasone	13	Mass effect/Perilesional edema in CNS	Improvement
Benard et al., 2012	4	44	M	2	Acute/Subacute	Lymph nodes	Hydrocortisone	21	Paradoxical inflammatory reaction	Improvement
		40	M	5	Chronic disseminated	Mucocutaneous	Prednisone	40	Ulcer	Improvement
						Larynx			Laryngeal involvement	
						Lymph nodes				
						Lungs			Paradoxical inflammatory reaction	
		38	M	8	Chronic disseminated	Larynx	Prednisone	40	Ulcer	Improvement
						Skin			Laryngeal involvement	
						Lymph nodes				
						Lungs			Paradoxical inflammatory reaction	
Guevara et al., 2024	1	9	M	3	Acute/Subacute	Lymph nodes	Prednisone	14	Paradoxical inflammatory reaction	Improvement
						Liver and Spleen				
Present Report	2	54	F	4	Acute/Subacute	Lymph nodes	Hydrocortisone	15	Paradoxical inflammatory reaction	Improvement
						Digestive				
		28	M	2	Acute/Subacute	Lymph nodes	Hydrocortisone	23	Paradoxical inflammatory reaction	Improvement
						Digestive				

The mechanisms underlying this inflammatory reaction in PCM patients remain unclear and may involve dysregulated, immune-mediated processes characterized by hypersensitivity reactions and/or exaggerated responses to persistent antigen release following fungal death^5^^,^^15^^,^^16^ This paradoxical reaction resembles IRIS observed in HIV-infected patients with advanced immunosuppression (CD4 < 100 cells/mm^3^), occurring several weeks or months after ART initiation^11^ Unlike tuberculosis and leprosy paradoxical reactions ‒ along with other conditions where corticosteroids are commonly used as adjunctive therapy based on clinical judgment or consensus ‒ their indication in PCM has been very limited due to the lack of robust data and reliance on analogy with other diseases^6^^,^^9^, ^10^, ^11^, ^12^, ^13^, ^14^

Due to longstanding concerns regarding the long-term adverse effects of corticosteroids, their use in patients with various infections has been restricted and remains controversial. However, evidence shows that they can improve survival, provide long-term benefits, and relieve diverse symptoms depending on the type of infection, without interfering with the effectiveness of antimicrobial therapy. Conversely, they may be ineffective or even harmful in specific situations^20^

In the context of PCM, we reviewed 21 clinical reports comprising 44 patients who received corticosteroids to alleviate symptoms related to compressive, obstructive, or inflammatory effects in various organs. Most patients had the severe acute/subacute clinical form and exhibited favorable outcomes. Although these reviewed data fall outside the scope of the present report and are not individually cited, they support the potential pivotal role of corticosteroids as adjunctive therapy in severe and life-threatening cases of PCM.

Although the true incidence of PCM is unknown, it is one of the most prevalent endemic mycoses in Latin America, and it is highly likely that additional unidentified cases with paradoxical inflammatory reactions have occurred over time and were mistakenly considered part of the disease’s natural progression. The hypothesis that this reaction resembles IRIS in HIV-infected patients is compelling and may provide insights into its pathophysiology, contributing to improved management strategies^6^^,^^11^

Thus, it is essential that infectious disease specialists, clinical mycologists, and other clinicians caring for PCM patients in endemic Latin American regions consider the current evidence supporting the clinical benefit of corticosteroids in selected and severe forms of PCM. Furthermore, it is highly desirable that similar cases be reported to better define the true occurrence of this paradoxical reaction and to clarify the role of corticosteroids as adjunctive therapy, thereby improving patient outcomes and strengthening the evidence base for this intervention.

Data availability statement

The data that support the findings of this study are available from the corresponding author upon reasonable request.

## Conflicts of interest

The authors declare no conflicts of interest.
